# Quantitative assessment of damage during MCET: a parametric study in a rodent model

**DOI:** 10.1186/s40349-015-0039-2

**Published:** 2015-10-16

**Authors:** Yiying I. Zhu, Douglas L. Miller, Chunyan Dou, Xiaofang Lu, Oliver D. Kripfgans

**Affiliations:** Department of Biomedical Engineering, University of Michigan, Ann Arbor, MI 48109 USA; Department of Radiology, University of Michigan Health System, Ann Arbor, MI 48109 USA

**Keywords:** Cavitation microlesions, Hypertrophic cardiomyopathy, Myocardial macrolesion, Therapeutic ultrasound, Quantitative therapy analysis

## Abstract

**Background:**

Myocardial cavitation-enabled therapy (MCET) has been proposed as a means to achieve minimally invasive myocardial reduction using ultrasound to produce scattered microlesions by cavitating contrast agent microbubbles.

**Methods:**

Rats were treated using burst mode focused ultrasound at 1.5 MHz center frequency and varying envelope and pressure amplitudes. Evans blue staining indicated lethal cardiomyocytic injury. A previously developed quantitative scheme, evaluating the histologic treatment results, provides an insightful analysis for MCET treatment parameters. Such include ultrasound exposure amplitude and pulse modulation, contrast agent dose, and infusion rate.

**Results:**

The quantitative method overcomes the limitation of visual scoring and works for a large dynamic range of treatment impact. Macrolesions are generated as an accumulation of probability driven microlesion formations. Macrolesions grow radially with radii from 0.1 to 1.6 mm as the ultrasound exposure amplitude (peak negative) increases from 2 to 4 MPa. To shorten treatment time, a swept beam was investigated and found to generate an acceptable macrolesion volume of about 40 μL for a single beam position.

**Conclusions:**

Ultrasound parameters and administration of microbubbles directly influence lesion characteristics such as microlesion density and macrolesion dimension. For lesion generation planning, control of MCET is crucial, especially when targeting larger pre-clinical models.

## Introduction

Hypertrophic cardiomyopathy (HCM) is a common genetic cardiovascular disease, which is usually clinically recognized by a maximal left ventricular wall thickness greater than 15 mm [1]. This globally prevalent disease, reported in about 0.2 % (i.e., 1:500) of the general population, is the most frequent cause of sudden death in young people and can lead to functional disability from heart failure and stroke [2].

The traditional treatment for HCM to reduce myocardium is septal myectomy. This surgical method removes septal hypertrophy, which possibly leads to perturbation of mitral valve leaflets [3]. An innovative therapeutic scheme, named myocardial cavitation-enabled therapy (MCET), has been proposed as a means to achieve minimally invasive myocardial reduction by cavitating contrast agent microbubbles with ultrasound to produce a fractional macrolesion containing sparse and histologically definable microlesions [4]. There are several ways of controlling cavitation here. Cavitation is enabled by the injection of ultrasound contrast agents. These will enable cavitation only in the focal region of the transducer and thus only there lead to microlesion formation in the myocardium. Second, ultrasound cavitation is dependent on sound pressure amplitude. In vivo experiments reveal that cavitation-induced lesions take place at peak rarefactional pressures larger than 2 MPa as obtained under free field conditions. In this case, ECG is monitored for premature complexes. It has been seen that the occurrence of premature complexes is directly correlated with cavitation events [5].

As a potential tissue reduction therapy, MCET avoids open-chest surgery and is hypothesized to allow healing with minimal scar formation, resulting in shrinkage of the cardiac treatment volume. This ultrasound microbubble-enabled method additionally provides the possibility of guiding and monitoring via quantifying feedback from the microbubble emissions.

To optimize MCET ultrasound parameters and administration of microbubble settings, assessment of the therapeutic effect is needed to assist parameter adjustment. Efforts in computerized analysis have been made to aid diagnostics and therapy for being fast, objective, and quantitative. Methods have been developed for computed tomographic angiography for the purposes of detecting heart diseases [6, 7] and for quantification of coronary arterial stenosis [8]. Automatic detection of pulmonary embolism has also been used in CT angiography [9, 10]. Quantitative ultrasound has been employed in diagnosis of osteoporosis [11], as well as in at-risk pregnancies with three-dimensional sonographic measurement of blood volume flow in umbilical cords [12]. Three-dimensional high-frequency ultrasound data also has been processed to offer a quantitative evaluation of cancerous lymph nodes at the microscopic level [13].

For MCET, a quantitative method for assessing the distribution and total accumulation of myocardial necrosis based on Evans blue-stained cells in the tissue histology slices was developed previously [14] and is used in this study. This paper investigates the tuning of various parameters involved in MCET and paves the way for pre-clinical treatment planning of myocardial lesion creation and properties thereof, in a quantitative manner.

One important and practical aspect of MCET is managing the buildup of microlesions and macrolesions to achieve a desired amount of myocardium reduction in larger pre-clinical models as well as, ultimately, in the clinic. Acoustic pressure amplitude, contrast dose, and treatment duration are adjustable variables. The parametric exploration of various conditions will assist in the search for feasible treatment conditions that allow for fast lesion creation with a 15–20 % microlesion density and a large axial and lateral dimension. Another desirable factor for practical clinical implementation is the treatment efficiency. Instead of treating a single focal spot as done in our previous study [4], a scanned beam would allow for a more rapid accumulation of lesions in a larger target treatment volume.

Our method of computer-aided histology analysis was developed using relatively high exposure parameters to reflect therapeutic treatment conditions [12]. This provided a means to reconstruct the tissue volume containing microlesions and their distribution, which can then be integrated to yield the potential fraction of tissue reduction. For validation, a visual scoring method was used in tandem, in which lethally injured cells indicated by fluorescent staining in frozen sections were counted. The visual method has been the gold standard for quantifying cell death by counting the absolute number of stained cells. However, when the number of stained cells becomes large, as for treatment (rather than exploring bioeffects), the visual method becomes a qualitative scoring method, which was suspected to yield inaccurate results for the validation for the computer-aided method. The purpose of this study was to analyze several exposure groups, which had reduced, sub-therapeutic treatment effects, using quantitative visual scoring for comparison to the computer-aided analysis.

## Materials and methods

### Experimental conditions

**Fig. 1:**
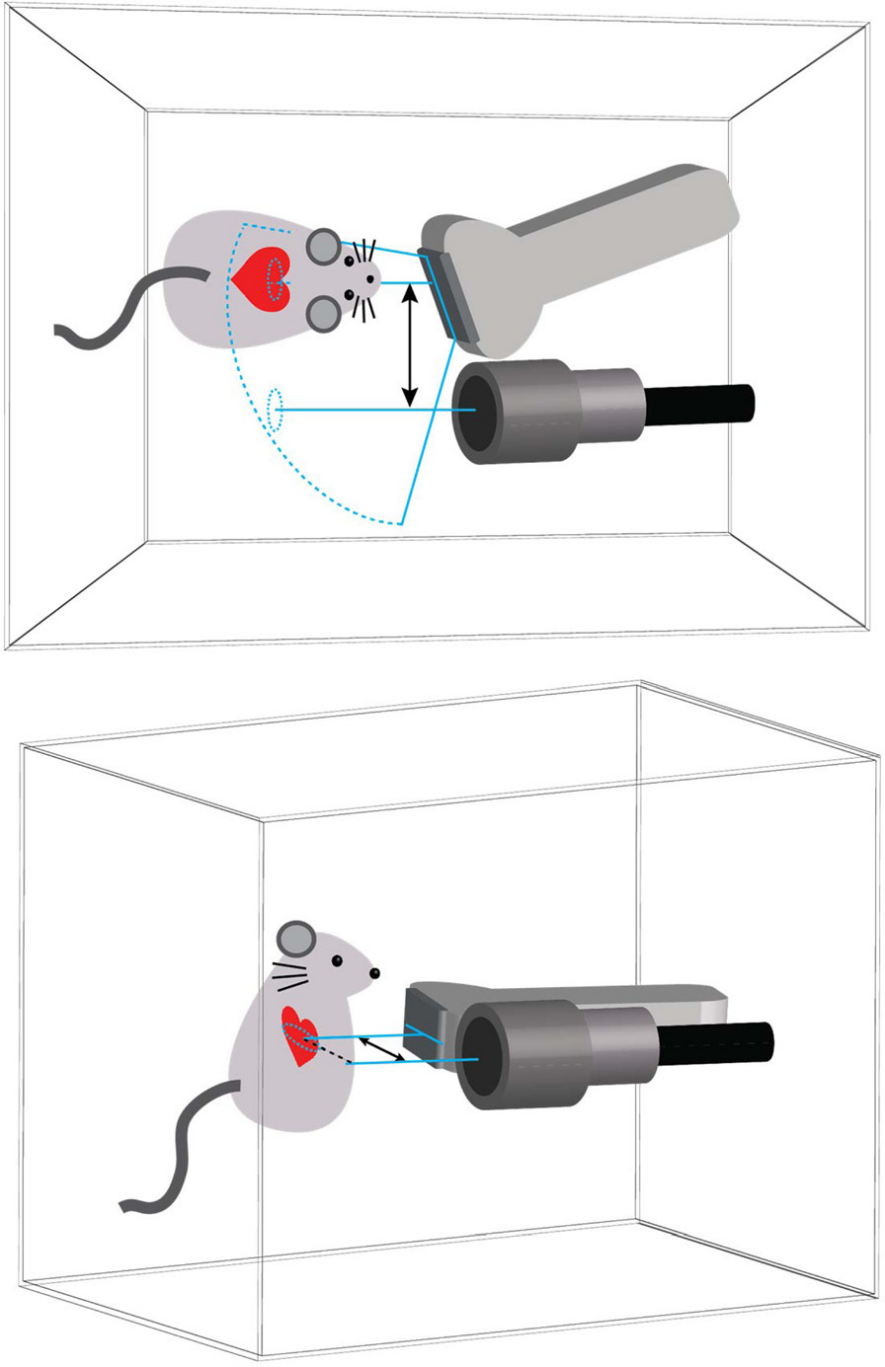
Schematic illustration (*top view* and *side view*) of the experimental setup. The imaging array and therapeutic transducer were fixed at a 37° angle so the acoustic axis of the therapy transducer was parallel with a specific image line of the sector array. This image line was identified in a water tank using a line target and marked on the screen of the ultrasound scanner. This image line was then used to warrant the acoustic access to the left ventricular wall. Subsequently, the transducer/probe gantry was translated so the therapy transducer beam followed the same path as for the previously identified image line

In order to make MCET amenable to clinical translation, the evaluation of B-mode echogenicity (more generally backscatter), physiological responses, i.e., premature complexes and visual scoring [15] is employed. For this study, tissue samples were collected and prepared for histological evaluation. Specifically, these samples underwent quantitative analyses aimed to assist the treatment planning. In vivo animal procedures were conducted on 50 male Sprague-Dawley rats (Charles River, Wilmington, MA, USA) including 5 sham rats weighing 352 ± 30 g. Approval and guidance of all animal work was done by the University Committee on Use and Care of Animals. All rats in the treated group were injected with Definity® (Lantheus Medical Imaging, Inc., N. Billerica, MA) at a rate of 5 or 12.5 μL/kg/min. Microbubbles suspended and diluted in sterile saline were infused via a tail vein or jugular vein catheter (gauge #24) starting 15 s before ultrasound exposure and concluding with the end of exposure. MCET was performed with ultrasound bursts of 5-cycle pulses at a center frequency of 1.5 MHz and a pulse repetition frequency of 4 kHz. The ultrasound exposure system consisted of a function generator for generating a pulse train (model 3314A function generator, Hewlett Packard Co., Palo Alto CA), an arbitrary waveform generator for amplitude modulation of the pulse train (model 33220A, Agilent Technologies, Loveland CO), a power amplifier (A-500, Electronic Navigation Industries, Rochester NY), and a 1.5-MHz single element therapy transducer (Panametrics A3464, Olympus, Waltham, MA). The therapy transducer was a standard single-element focused transducer, with a 1.9-cm diameter and 3.8-cm focal length. The treatment was targeted with the aid of diagnostic ultrasound imaging (GE Vivid 7 with S-10 phased array, GE Healthcare, Jupiter FL, USA) operated at 10 MHz with a 5-cm focal depth, as previously described [15]. The setup scheme as illustrated in Fig. 1 provided targeting of the therapy beam and for low-power imaging of the heart during exposure. The imaging array and therapeutic transducer were fixed at a 37° angle such that the acoustic axis of the therapy transducer is parallel with one image line of the sector array. This image line was identified in a water tank using a line target and marked on the screen of the ultrasound scanner. Then, this image line was used to evaluate acoustic access to the left ventricular wall. Subsequently, the transducer/probe gantry was translated such that the therapy transducer beam aimed along the same fixed path as the image line identified in the image to pass through the window between the ribs (vertical axis) and between the sternum and the left lung. Burst emissions were triggered from the ECG signal at every four heartbeats end-systole. Prior to ultrasound exposure, all rats were injected with Evans blue, a reliable histological stain for lethal injury of cardiomyocytes [15].

The study was conducted with groups of five rats each and given letter designations to identify the specific set of conditions in each group. Groups A to J were designed specifically to test treatment effects for different parameters, as listed in Table 1. Except for groups G and H, all rats were exposed to ultrasound with a maximum rarefactional pressure amplitude (PRPA) of 4 MPa. The pulses in the center of the focal region were measured in a water bath using a calibrated hydrophone with a 0.2-mm diameter aperture (model HMA-0200, Onda Corp., Sunnyvale, CA) to acquire the point spread function and the electro-acoustic transfer function. Comparison of groups A and B tested the influence of catheter placement, with the jugular vein giving a central and larger vein access than the tail vein. Groups B and C were compared to test the effect of aiming ultrasound near the ribs. Those two comparisons were performed intentionally to test experimental perturbations.

**Table 1:** Table of sets of conditions used for respective groups of rats, with a cohort of five animals each

Experiment conditions for rat groups					
Group ID	Infusion site	Pressure (PRPA)	Modulation	Infusion rate	Treatment duration
A	Tail	4 MPa	Square	5 μL/kg/min	5 min
B	Jugular	4 MPa	Square	5 μL/kg/min	5 min
C	Jugular	4 MPa	Square	5 μL/kg/min	5 min
D	Jugular	4 MPa	Gaussian	5 μL/kg/min	5 min
E	Jugular	4 MPa	Gaussian	12.5 μL/kg/min	2 min
G	Tail	2 MPa	Gaussian	5 μL/kg/min	5 min
H	Tail	2.8 MPa	Gaussian	5 μL/kg/min	5 min
I	Tail	4 MPa	Gaussian	5 μL/kg/min	100 s
J	Tail	4 MPa	Gaussian	5 μL/kg/min	30 s

Group F acted as a sham and calibration group, as described in the text

**Fig. 2:**
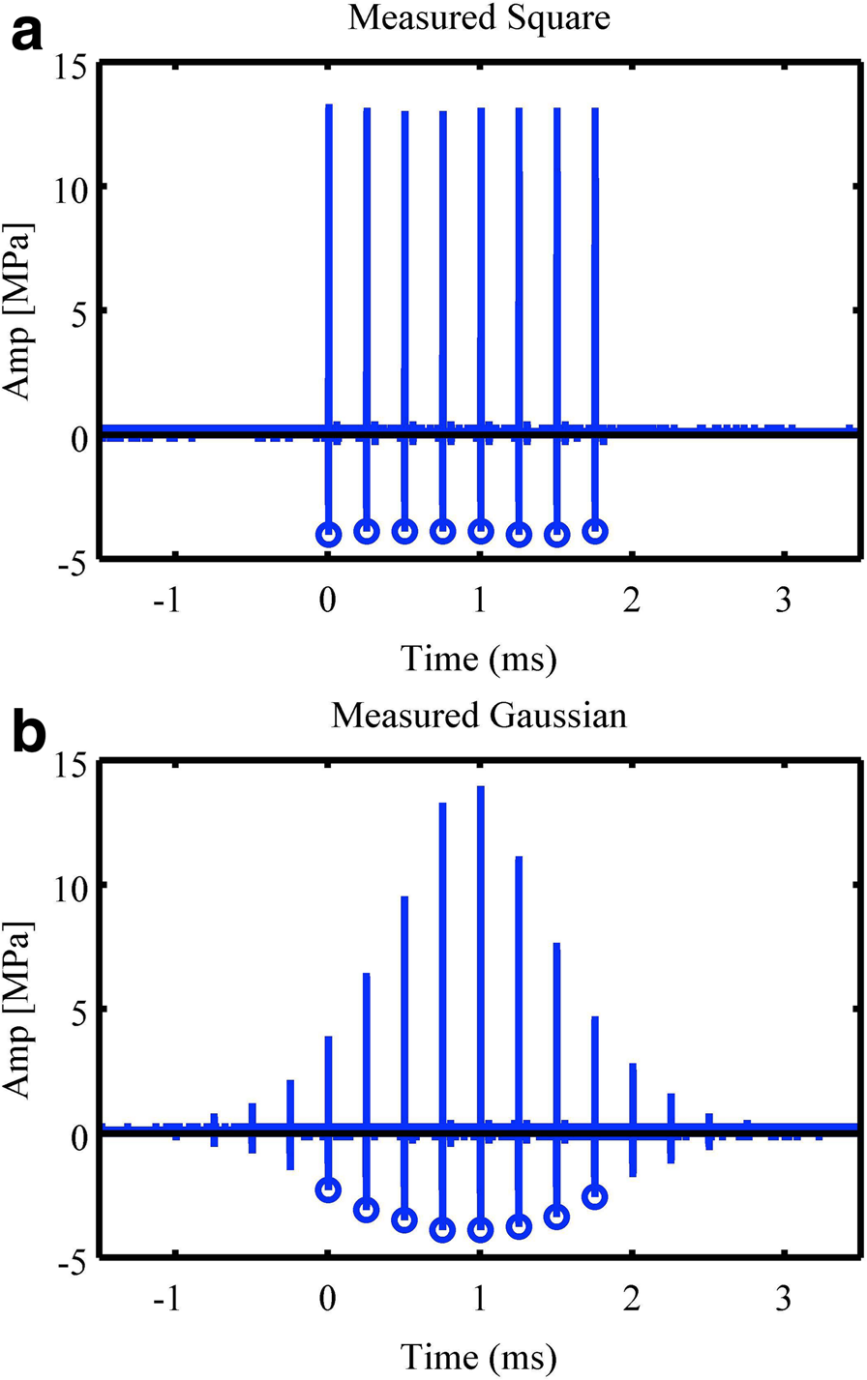
Employed electric pulse train excitations. *Circles* in square modulation (**a**) and Gaussian modulation (**b**) pulse trains indicate the employed negative pressure amplitudes. Note that each spark is a 5-cycle 1.5 MHz tone burst

Groups B and D compared the results of treatment using square versus Gaussian pulse train modulations of the acoustic pressure. The single element transducer delivered a Gaussian envelope pulse train, such that the full-width half maximum of the Gaussian modulation was 2 ms. This modulation scheme was to simulate an exposure that would be experienced by contrast agent in the presence of a sweeping ultrasound beam, as found in a diagnostic imaging setup [16]. To reduce treatment times for larger treatment, volumes such a beam could be implemented as a sweeping therapy beam and is thus included in the tested exposure conditions. Note that only the pulse train envelope is Gaussian modulated. Each individual pulse is a constant amplitude 5-cycle tone burst (see Fig. 2). The amplitude modulation was set to give zero exposure unless a modulation envelope signal was triggered. The envelope signal was either a 2-ms square pulse with a constant amplitude of 4 MPa PRPA or a Gaussian modulation function that produced a 2-ms pulse with amplitudes greater than 2 MPa PRPA. The Gaussian modulation was designed to emulate a scanned ultrasound beam from a clinical ultrasound scanner, with approximately 56 frames per second (fps).

Different contrast agent dose rates were tested by comparing groups D and E. The previous rate was 5 μL/kg/min, representing the recommended dose for diagnostic applications [4]. A higher infusion rate of 12.5 μL/kg/min was tested for the possibility of using a higher dose in therapeutic applications, which may reduce treatment durations.

Comparison between groups G, H, and D evaluated the dependence of lesion formation on acoustic pressure. The three groups were respectively exposed to ultrasound fields of 2, 2.8, and 4 MPa PRPA. Groups J, I, and D, on the other hand, evaluated microlesion accumulation by varying the treatment duration, i.e., adjusting the contrast infusion duration. Groups G, H, I, and J were specifically treated with sub-therapeutic parameters with reduced treatment impact on cell survival. Correlation between acoustic pressure, treatment duration, and induced microlesion density was intended to establish some dynamic range for microlesion induction.

Finally, group F was a sham and calibration control group, in which each rat received the full 4 MPa therapy exposure before the contrast agent infusion started.

Results in groups are presented in boxplots. For each box, the central mark is the median, the edges of the box are the 25th and 75th percentiles, the whiskers extend to the most extreme data points not considered outliers, and outliers are plotted individually. The normal range was defined as *q*_3_ + 1.5 (*q*_3_ − *q*_1_) or smaller than *q*_1_ − 1.5 (*q*_3_ − *q*_1_), where *q*_1_ and *q*_3_ are the 25th and 75th percentiles, respectively.

### Cardiomyocyte scoring

Rat hearts were harvested and scored 1 day after exposure as described in previous work [15]. Briefly, up to 40 10-μm-thick frozen sections were made from the treated volume in each heart. A quantitative method for assessing the distribution and total accumulation of myocardial necrosis is based on Evans blue staining and was developed previously [14]. Microlesions were identified by fluorescence microscopy and photographs of each section. Image registration was then performed to digitally stack the frozen sections in 3D and to reconstruct a model of the heart morphology in the entire sampled region showing the three-dimensional distribution of microlesions. The microlesion fraction of the tissue within the focal zone was calculated to estimate the potential fractional volume of tissue reduction that was achieved. Quantitative results were characterized in terms of microlesion volume, macrolesion volume, microlesion lesion density, and dimensions of the radially symmetric approximated macrolesion.

In addition to the computer-aided assessment, traditional visual scoring was used to evaluate myocardial necrosis qualitatively by visual identification and quantitatively by scoring of Evans blue-stained cells using fluorescence microscopy [15]. Automatic scores were obtained from dividing the geometric microlesion volume by a constant conversion factor acquired from a geometry-based cardiomyocyte model [14].

### Therapeutic field simulation

**Fig. 3:**
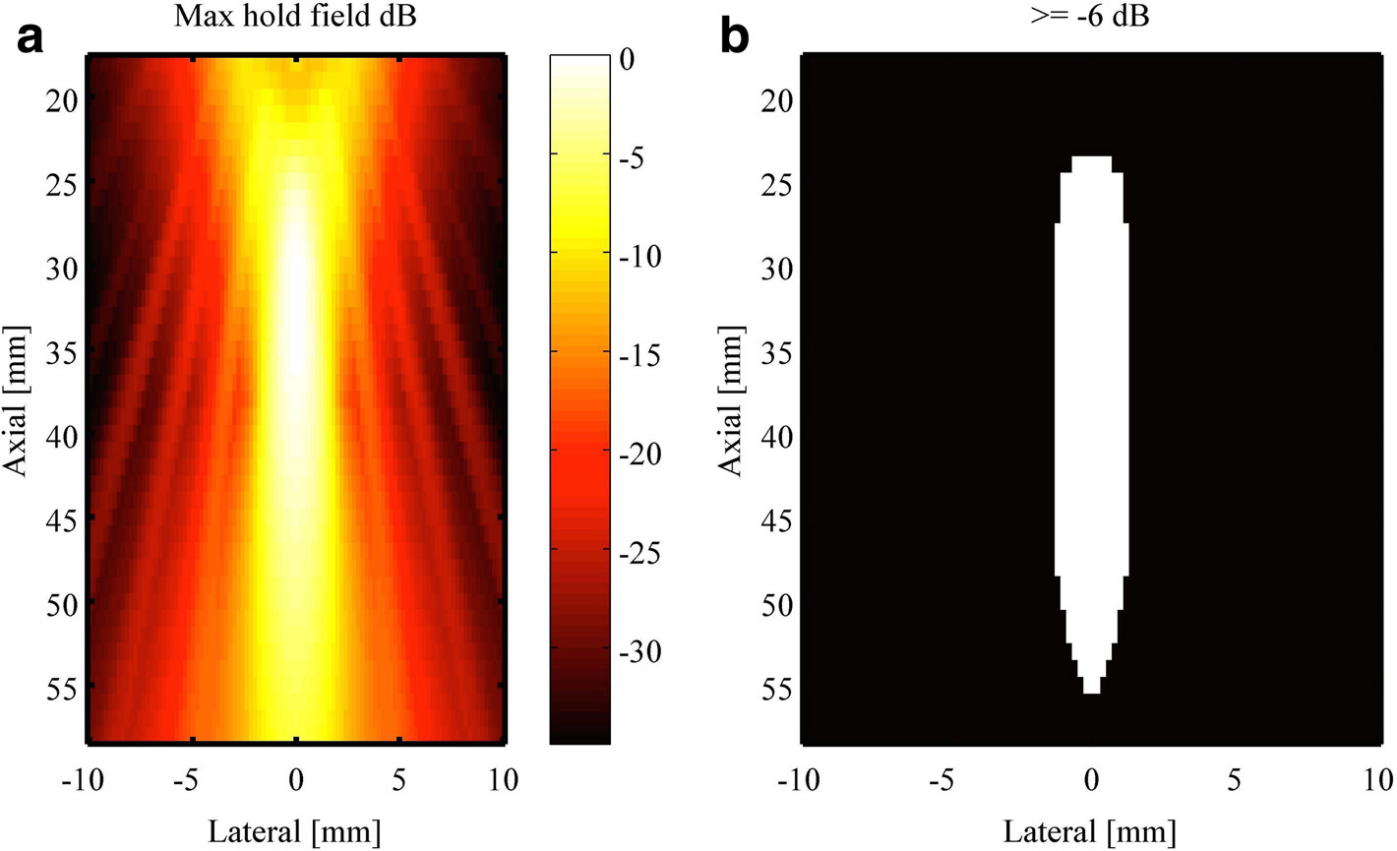
Therapeutic ultrasound transducer field simulation. **a** FIELD II simulation of pressure distribution for free field single-element therapy transducer. **b** Lesion formation based on the lesion-pressure dependence; volume narrows the region of lesion formation to where peak negative pressure is above 2 MPa, i.e., greater than the lesion formation pressure threshold. The acoustic pressure field representing the expected lesion region is 2.5 × 32 mm at the level of −6 dB relative to 4 MPa

The acoustic field, assuming a water path, for the employed single-element therapeutic transducer was simulated in FIELD II [17], a widely used ultrasound simulation program. A 1.9-cm diameter concave single-element transducer with 3.8 cm focus excited at 1.5 MHz with a 5-cycle burst was modeled. Two successive bursts 250 μs apart (4 kHz PRF) were significantly larger than the 3-μs pulse train length. Thus, they were considered to have no interference on each other’s acoustic field. Figure 3a shows a normalized field reflecting the maximum exposed pressure during one burst. The effective region of sound pressures with amplitudes above the acoustic pressure threshold of 2 MPa is indicated in Fig. 3b. By revolving this shown region along the lateral (*x* = 0 mm) axis, the effective volume, denoted as an axisymmetric rotational model, was calculated. This simulation intended to associate in situ acoustic field with the formed lesion.

## Results

### Quantitative computer-guided lesion analysis

**Fig. 4:**
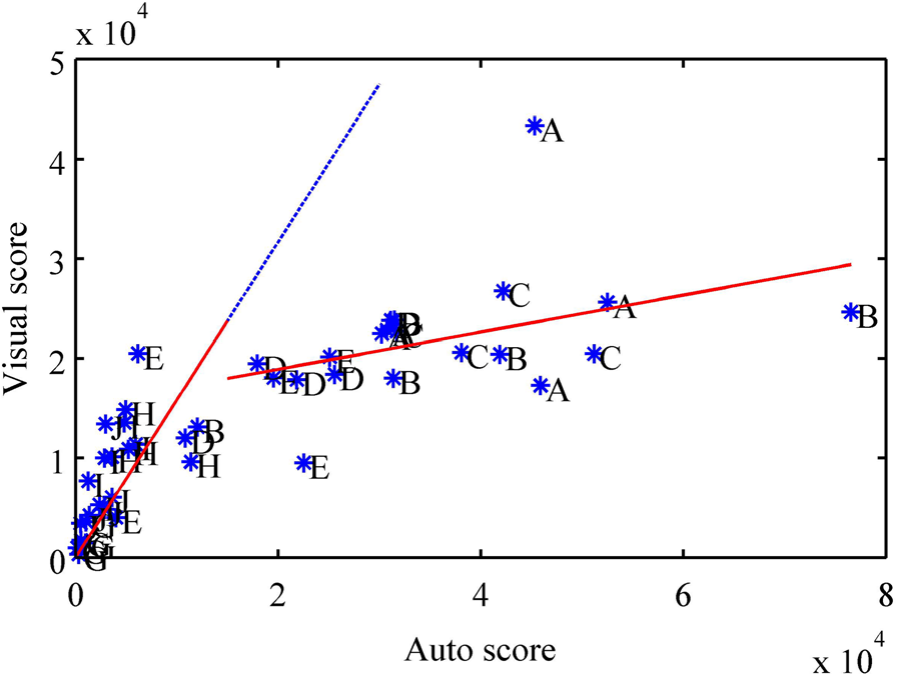
Scatter plot showing comparison of visual score versus auto score. The auto score was obtained from a computer-aided method [14] with piece-wise least square fitting at a break point for the auto score equal to 15,000. Visual scoring correlates best with auto scoring for fewer lesion counts, which reveals the difficulty to assess large number of lesions quantitatively by visual means

The previous study [4] was limited by a low dynamic range with respect to the number of induced lesions. Current results span a large range of number of lesions per area on histology, which therefore allows for a more critical evaluation of the performance comparison between visual and automatic scoring. Data presented in this study reach to tens of thousands of lesions, a range that is difficult to assess quantitatively by traditional visual scoring. Figure 4 shows visual scores plotted versus auto scores (obtained from computer-aided algorithm) for all groups listed in Table 1. The data can be segmented in two regions: low counts ranging from 0 to 15,000 and high counts from 15,000 to 80,000 (both with respect to the auto score), respectively. A linear least square fit of the form *y* = *a*_1_·*x*, was performed for the low lesion count segment and is shown in red and extended as blue in Fig. 4. Another linear least square fit, now of the form *y* = *a*_2_·*x* + *b*, was performed for the high lesion count segment and is shown in red in Fig. 4. Best-fit coefficients were found as: *a*_1_ = 1.52, *a*_2_ = *0.19* and *b* = 15,216, with *a*_1_ greater than 1 would mean that visual scoring counted more cells, *a*_2_ being so low shows that visual scoring counted groups of cells as one. This result clearly showed the “saturation” effect of the visual scoring method for high cell counts.

### Acoustic pressure dependence

#### Macrolesion volumes

**Fig. 5:**
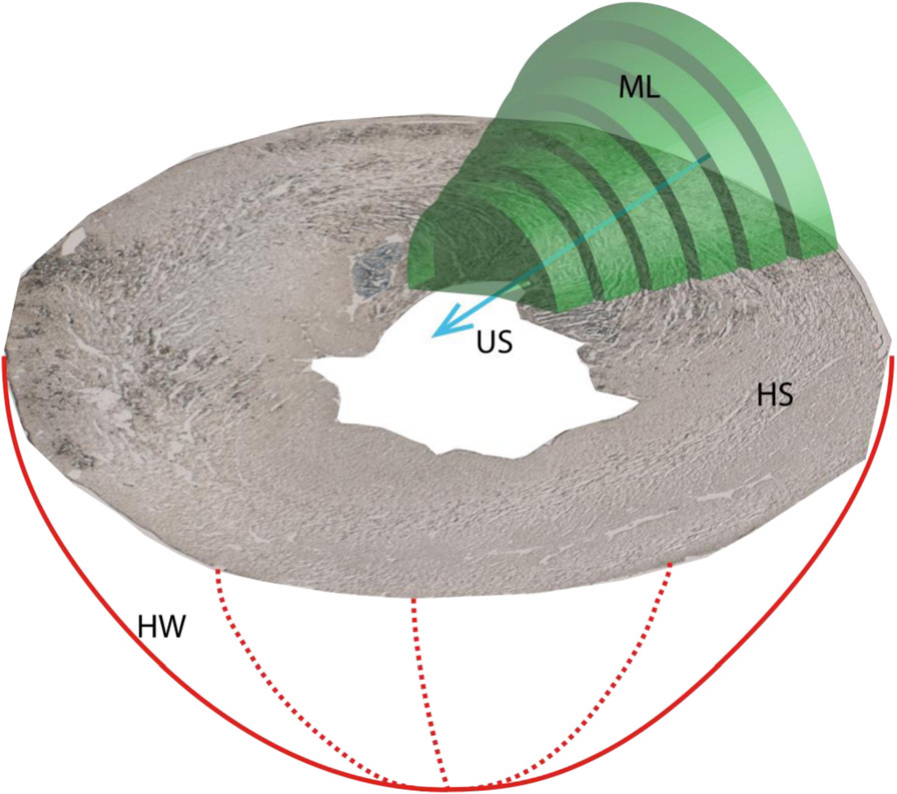
Diagram illustrating a macrolesion as characterized from a stack of histology slices. The heart wall (*HW*) is depicted in *red lines*. A cross section of the heart is shown as a histological slice (*HS*), stained with Evans blue indicating cell necrosis, i.e., microlesions. The ultrasound (*US*) beam is indicated as a *blue line* and *arrow* and was derived from least square fitting of microlesions. Along the beam, the shown *green cylindrical disks* were characterized from cylindrical volume elements that contain 95 % of the local microlesions. These coaxially stacked disks form a radially symmetric volume, called the macrolesion (*ML*), which is assumed to correlate with the *in situ* acoustic field

**Fig. 6:**
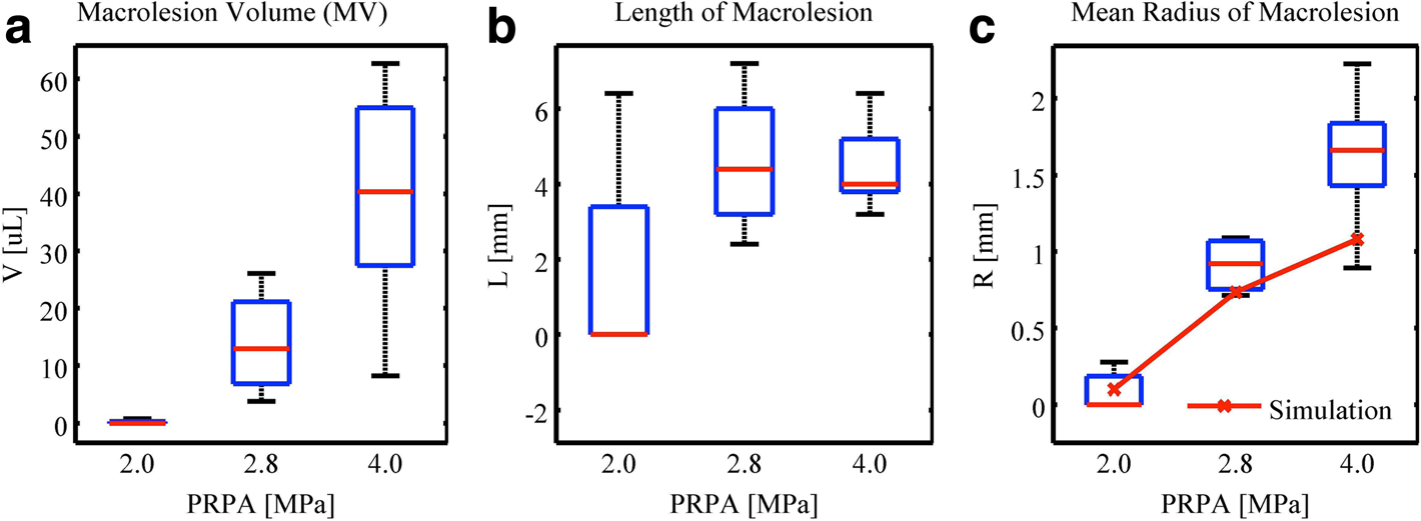
Treatment impact for different acoustic pressures. Here included **a** macrolesion volume, **b** length of macrolesion, and **c** radius of macrolesion for groups exposed under 2.0 MPa (G), 2.8 MPa (H), and 4.0 MPa (D). Simulations were done for a mean radius of acoustic field above the lesion formation pressure threshold of 2.0 MPa (*PRPA*). Macrolesion volumes appear positively related to acoustic pressure, where contributions come from the radial direction while the axial sizes are insignificantly different

Macrolesion dimensions are in part dictated by the therapy beam geometry as illustrated in Fig. 5. Lesion lengths (axial) and lesion diameters (lateral and elevational) are determined by the point spread function of the therapy transducer and the acoustic pressure of the transmitted wave. In other words, the volumetric region of the acoustic wave that bears pressure amplitudes above the pressure *p*_L_ required for lesion formation, will contribute to the therapy. This volumetric region grows as the pressure amplitude at the focus grows, and thus, larger acoustic pressures yield larger lesion count. Macrolesion dimensions are also dictated by the spatial availability of cells and contrast agent. The simulation gave an acoustic field of 2.5 × 32 mm at the level of −6 dB relative to 4 MPa as shown in Fig. 3b, representing the expected lesion region. In vivo results for the parametric acoustic pressure amplitude cases 2.0 (G), 2.8 (H), and 4.0 MPa (D) are illustrated in Fig. 6 using boxplots. Frequently, the axial dimension was limited by the thickness of the myocardium with the therapy beam penetrating the left ventricle entirely before the pressure amplitude fell below the threshold *p*_L_. Likewise, the therapy beam pressure rose above *p*_L_ before entering the myocardium. Therefore, an increase in pressure at the focus will only show limited increase in the axial lesion size. Radial macrolesion expansion is hyperlinear and follows the prediction of the simulation shown as the plotted curve in Fig. 6c. Simulations were done to compute the average radius of the acoustic beam above the hypothesized threshold *p*_L_ (2.0 MPa).

#### Microlesion characteristics

**Fig. 7:**
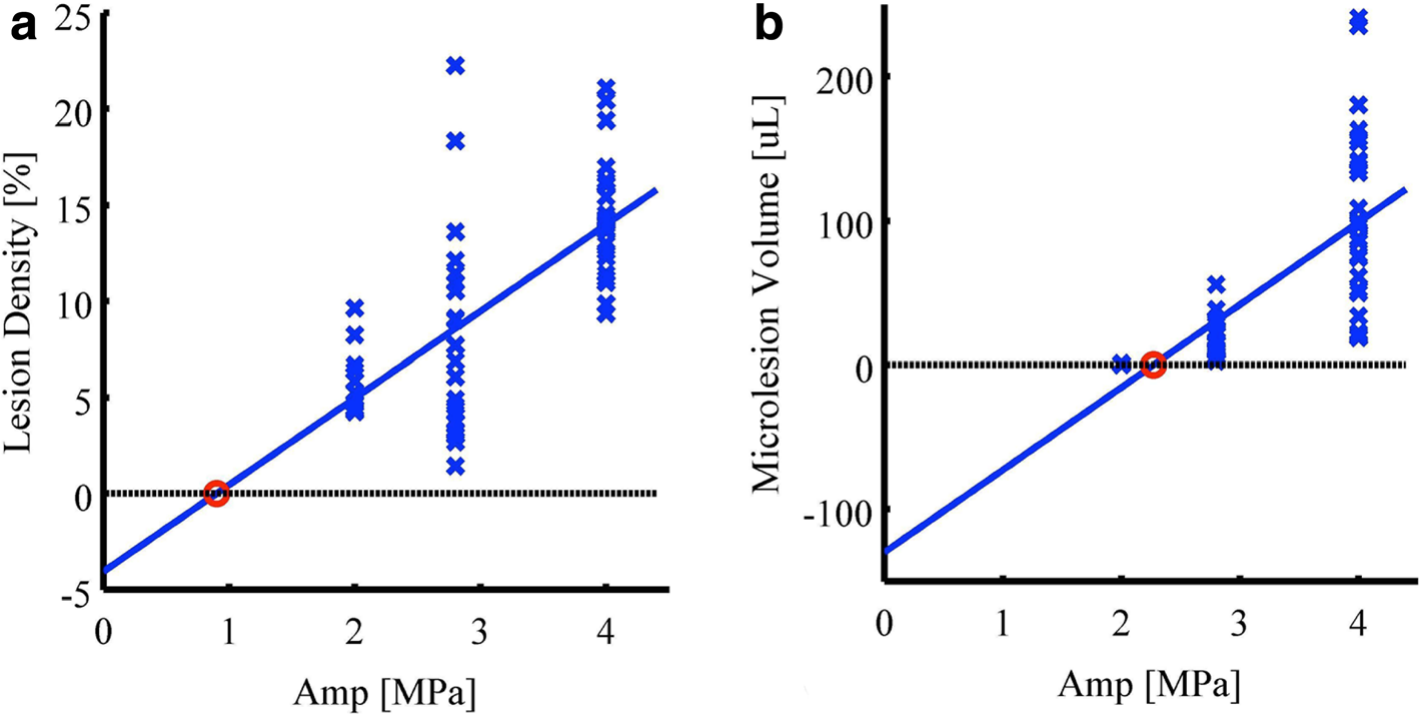
Treatment impact characterized for re-sliced volume along therapeutic beam. Results for exposures of 2.0, 2.8, and 4.0 MPa PRPA are shown for **a** scatter plot of lesion density with least square fitting and zero crossing 0.9 MPa PRPA and **b** scatter plot of microlesion volume with least square fitting and zero crossing 2.3 MPa PRPA

The spatial variation in lesion density was analyzed with respect to the therapeutic beam geometry. Specifically, lesion density was plotted as a function of local pressure amplitudes. Reslicing of the therapeutic beam volume was facilitated by placing axially oriented disks. Stacking them along the therapeutic beam as illustrated in Fig. 5 in green, allowed for plotting of lesion density as a function of local in situ pressure amplitudes. Least square fitting shown as a blue line in Fig. 7a resulted in *y* = 4.52*x* − 4.07, with 0 % lesion density occurring at 0.9 MPa indicated by the red circle. The microlesion volumes for resliced disks versus different acoustic amplitudes are plotted in Fig. 7b with least square fitting resulting in *y* = 57.2*x* − 130.0 and zero microlesion volume occurring at 2.3 MPa indicated by the red circle.

### Contrast agent availability versus macrolesion characteristics

As alluded to in the previous section, macrolesion dimensions are dictated by several experimental conditions, including contrast agent availability. More available agent will likely generate more lesions. On the other hand, more contrast agent per unit time may lead to agent-induced acoustic shadowing and a diminished in situ pressure wave amplitude. Results for changes in contrast agent availability are shown next.

#### Infusion duration

**Fig. 8:**
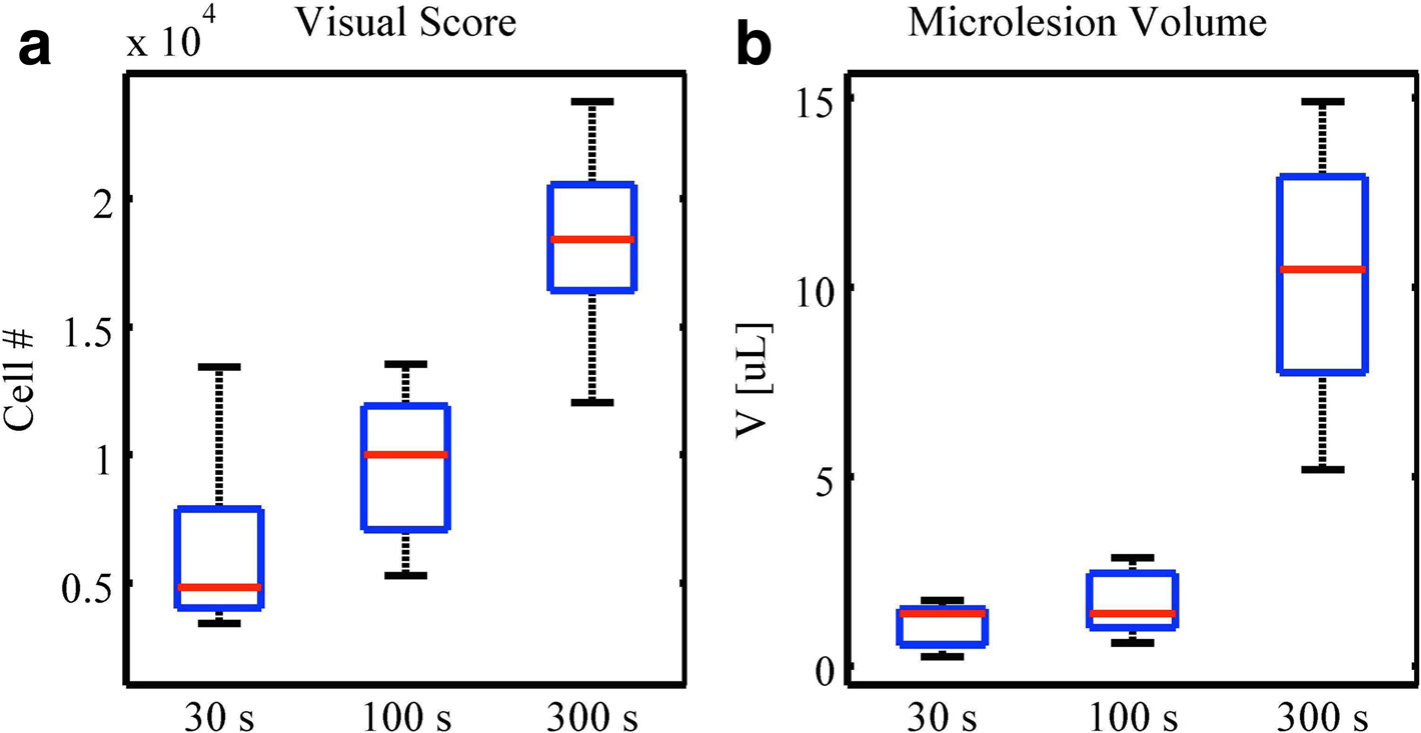
Treatment impact for different contrast agent infusion duration. Results for **a** visual score and **b** microlesion volume for groups infused with contrast agents for 30, 100, and 300 s, at a rate of 5 μL/kg/min, for groups J, I, and D, respectively. A positively correlated number of generated lesions are seen for the respective total infusion duration. Differences in visual score and microlesion volume for durations of 30 and 100 s are not statistically different. However, an infusion duration of 300 s significantly differs from the former two conditions

At first, a constant infusion rate of 5 μL/kg/min was tested for three infusion durations, namely 30, 100, and 300 s, for groups J, I, and D, respectively. Under ultrasound exposure, increase in contrast infusion time allows for higher deposition of total number of microbubbles. Figure 8 shows a positively correlated number of generated lesions for the respective total infusion duration. Differences in visual score and microlesion volume for durations of 30 and 100 s are not statistically different. However, a 300-s infusion duration shows a significant increase relative to the former two conditions with respect to the characterized microlesion volume. Specifically, the microlesion volume that increases from 10, to 30, to 300 s is not linear and will be addressed in the “Discussion” section.

#### Infusion rate

**Fig. 9:**
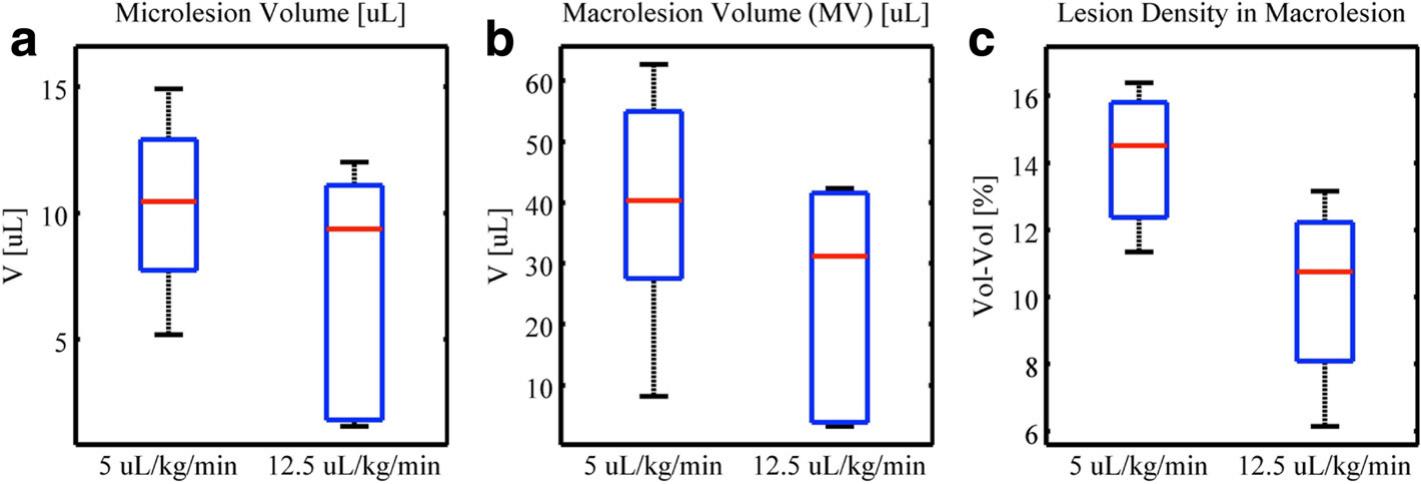
Treatment impact for different contrast agent infusion rates. Results are shown for **a** microlesion volume, **b** macrolesion volume, and **c** lesion density for groups infused with contrast agents for 5 min at a rate of 5 μL/kg/min (D) and 2 min at 12.5 μL/kg/min (E), i.e., yielding the same total dose. They are not significantly different, showing that the impact is dominated by the cumulative dose

Here, a constant total dose of contrast agent (25 μL/kg) was tested for two infusion rates, namely 5 μL/kg/min for 5 min and 12.5 μL/kg/min for 2 min, for groups D and E, respectively. A higher infusion rate, i.e., more agent per unit time, may lead to agent-induced acoustic shadowing and a diminished in situ wave pressure amplitude. Figure 9 shows no significantly different number of generated lesions for the respective infusion rates. Note that *microlesion volume* here refers to the total volume of all induced microlesions over the entire myocardium, which is not identical to the shown microlesion volume within the characterized macrolesion (macrolesion volume times lesion density).

### Therapy beam sweeping

**Fig. 10:**
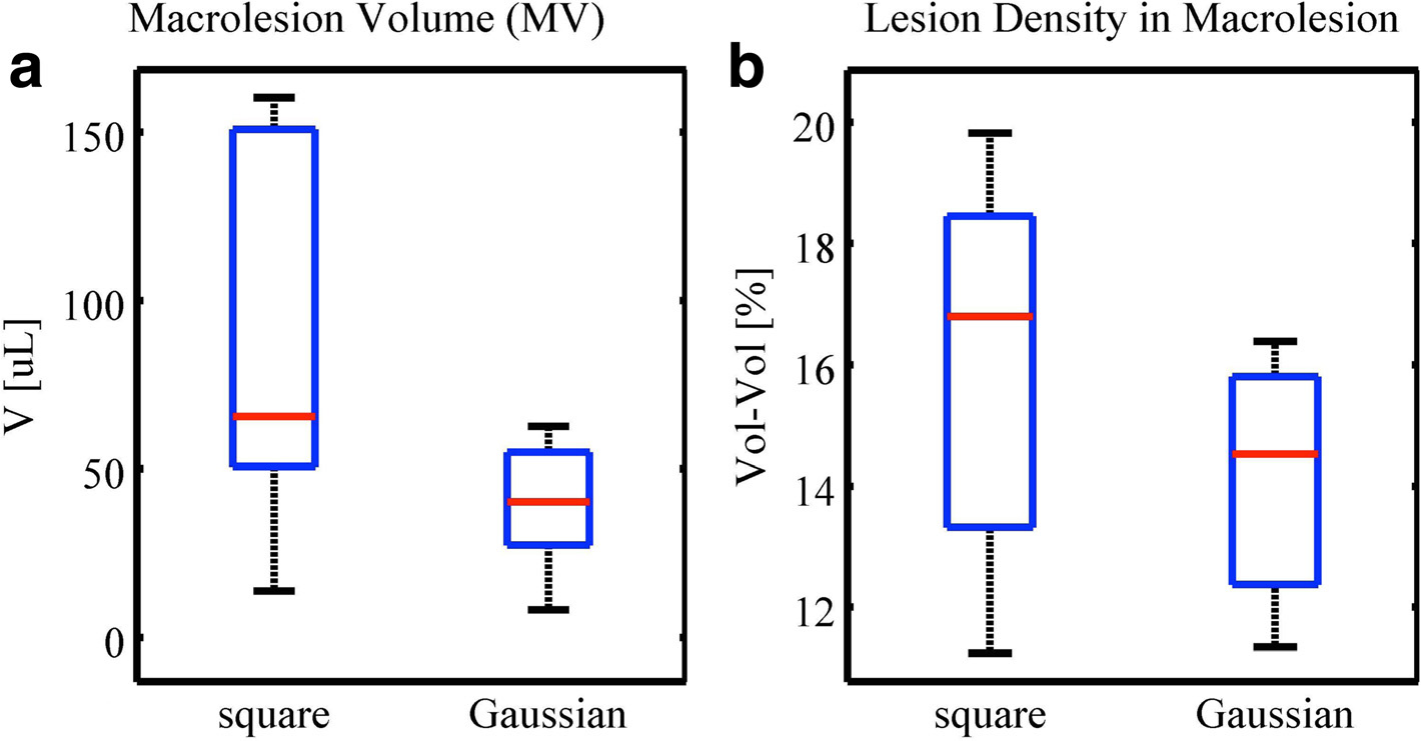
Treatment impact for square and Gaussian pulse modulation schemes. Results for the impact of individual focal treatment (group B) versus a swept beam (group D) are shown for **a** the corresponding macrolesion volume and **b** the observed lesion density. Similar impact is seen between Gaussian modulation and square modulation, which validates that a swept beam scheme might be a viable method to shorten treatment duration

In this experiment, Gaussian modulation was employed to mimic therapy beam sweeping. It was observed as shown in Fig. 10 that both the resulting macrolesion volume as well as the microlesion density inside the created macrolesion is reduced in comparison to the static beam, but not significantly different. The median macrolesion volumes resulting from square and Gaussian modulations are 65.6 and 40.3 μL respectively, and the median lesion densities are 16.8 and 14.4 %.

The pulse modulation groups corresponding to square and Gaussian profile are shown in Fig. 2. In the simulation modeling, the volume exposed by negative pressures greater than 2 MPa, and marked by circles, were integrated across space and time yielding 4.5 and 2.7 μL·s for square and Gaussian modulations, respectively.

## Discussion

The quantitative results generated by the previously developed and here tested computer-aided scheme provide possibilities for numeric and quantitative 3D lesion analysis and their dependence on experimental parameters that were investigated for their relevance for developing and improving MCET.

### Cardiomyocyte scoring

**Fig. 11:**
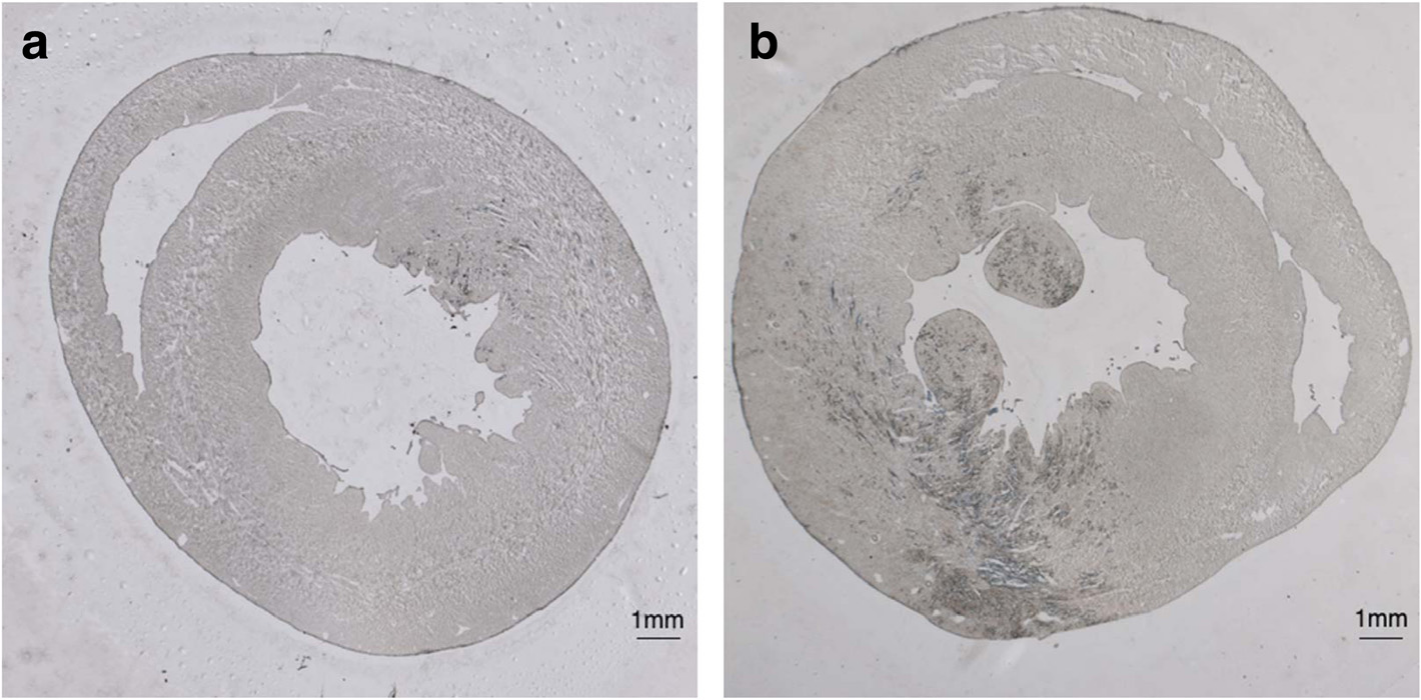
Example microscopic brightfield images of treated heart slices. The histology slice **a** contains sparse microlesions. Note that microlesions appear with a light blue stained by Evans blue. The shown histology violates the standard heart model shown in **b**. Distributed myocytes are lying in different tissue layers and with varying orientations

Visual lesion counting, as a traditional evaluation method, is appropriate for low lesion count cases as discussed for Fig. 4. However, for therapeutic applications, a large number of lesions require tedious counting. The computer-aided scheme [14] provides for an objective and quantitative lesion analysis. As shown in Fig. 4, a dramatic change in visual score versus auto score for high-count cases opposed to low-count cases reveals the limitation of visual scoring, when counting large numbers of lesions. Machine-operated and tested algorithms prevail. Note that even at low counts, auto score has a positively correlated relationship to visual score but does not have a one-to-one correspondence. Variance arises from manual bias differentiating individual cells from a clustered cell pool. On the other side as for auto scoring, imperfection exists when a constant conversion factor is employed for converting microlesion area, i.e., image pixels, on a given histology slide to a fixed number of cells, Eq. 1. An additional limitation is the assumption that cells possessing the same dimensions for each heart layer but different orientations for three layers [14]. Our model uses a statistical average based on a standard heart. This might lead to partial violations when the actual geometry (Fig. 11a) differs from the standard heart (Fig. 11b).

### Experimental perturbations exclusions

**Fig. 12:**
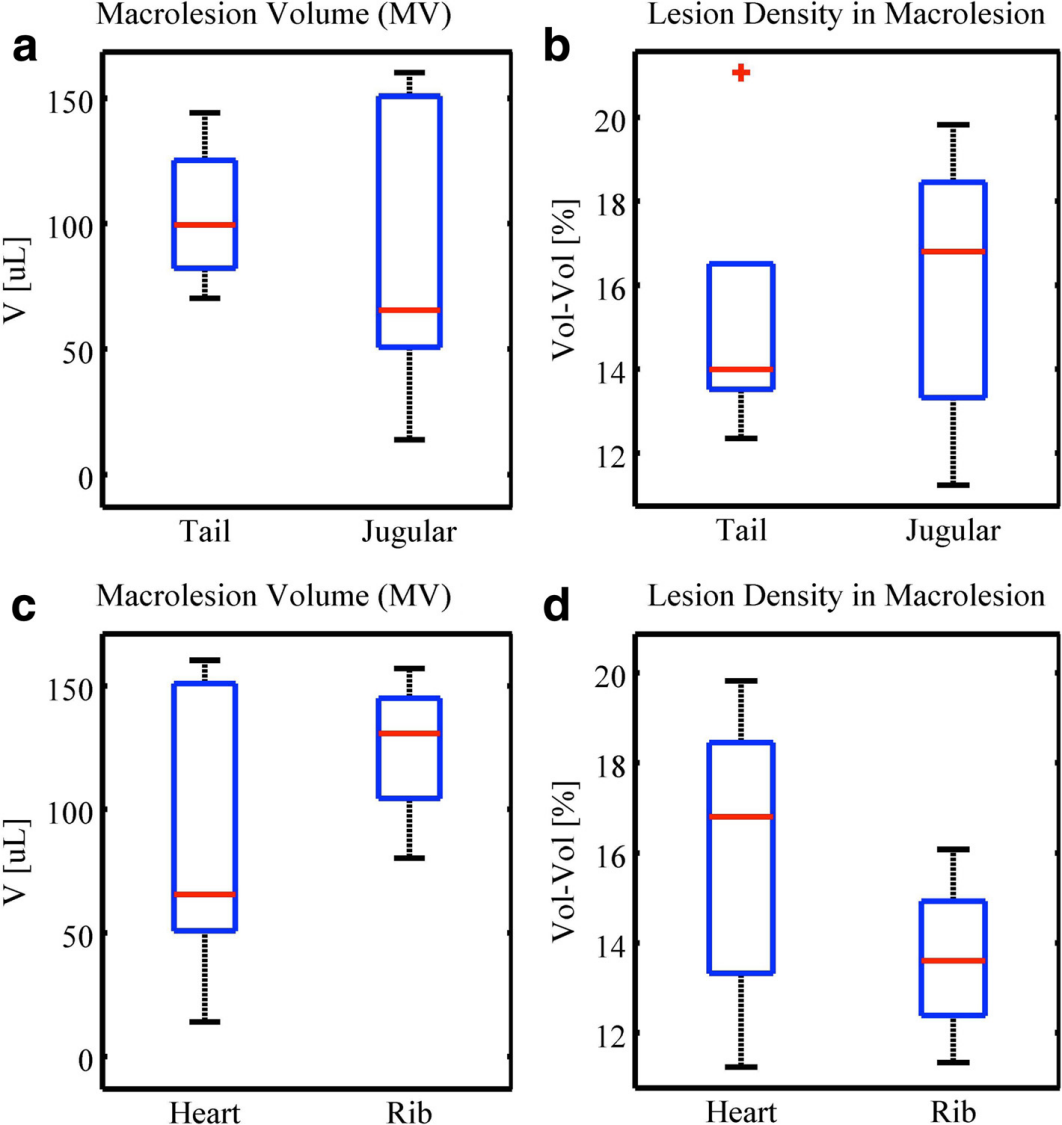
Comparison of treatment impact for different injections sites and ultrasound aiming strategies. Results show **a** macrolesion volume **b** and lesion density for two microbubble administration routes, i.e., tail vein (A) versus jugular vein (B) injection; additionally shown are **c** macrolesion volume and **d** lesion density for regular aiming (B) and intentionally aiming at the ribs (C). Injection and aiming variations do not significantly differ among the tested groups

Initially, the choice of injections sites, i.e., the jugular vein versus the tail vein, were thought to possibly influence the number of systemically circulating microbubbles. Comparing the two cohorts, however, showed that the two microbubble administration routes were equivalent. As shown in Fig. 12, tail vein (a) versus jugular vein (b) injection did not lead to significant differences in the subsequent lesion formation in either lesion density or in macrolesion volume. Additionally, variations of transducer aiming were tested. Intentionally aiming the ultrasound at the ribs (Group C) *does not* impact the therapeutic result. In vivo variations can be significant (see error bars in Fig. 12); however, there is a significant overlap between the tested routes for injection and between various aiming paths.

### Acoustic field modeling

#### Acoustic amplitude

Acoustic modeling provides a way to approximate lesion formation. In the therapy, impact analysis bioeffects of various acoustic exposures were investigated. The associated field simulation underestimates the mean radius of the macrolesion at 4 MPa as shown in Fig. 6c. This is because in vivo, some of the beam penetrates the left ventricle. This part of the beam was not excluded in the simulation; thus, the simulation resulted in averaging out the affected macrolesion radius. Another factor contributing to the greater treatment effect seen in vivo than the simulation comes from deformation of hearts after being harvested. Rats were treated at the ends of systole but hearts were relaxed after being sacrificed. Thus, the acoustic pattern may be distorted to some extent.

#### Swept beam

Treatment in humans will require focusing of the therapeutic beam at a larger area than currently done in rodents (rats). Such will either lead to the need of a modified, i.e., larger, point spread function or more likely numerous repetitions of individual exposures. The latter can be realized by either individual focal treatments or by employing a swept beam. The former will require a longer time for treatment of an equivalent total count of focal spots since the beam will be stepped from treatment location *n* to *n* + 1; therefore, a swept beam was investigated. Illustrated by “O” plot marks in Fig. 2 are the individual tone bursts that exceed the lesion formation pressure threshold. With that and the previously mentioned axisymmetric rotational model (point spread function), an effective treatment volume was simulated. Integration across space and time, yielded 4.5 and 2.7 μL·s for square and Gaussian modulations, respectively. Therefore, the swept beam simulation predicts an effective volume of 59.8 % of that of an individual focal treatment. The experimental macrolesion volume shown in Fig. 10a yields an effective median volume fraction of 61.5 %, supporting the axisymmetric rotational volumetric model.

### Thresholded induced and statistically accumulated lesion

#### Lesion formation as accumulated statistical events

In the experiment of increasing infusion, the slightly decreasing trend of lesion density shown in Fig. 6 may indicate some shadowing effect caused by a large population of instantaneous microbubbles placed along the beam path, acting as scatterers. However, the shadowing factor will need to be verified for the cases of a longer path, such as in a larger animal model.

**Fig. 13:**
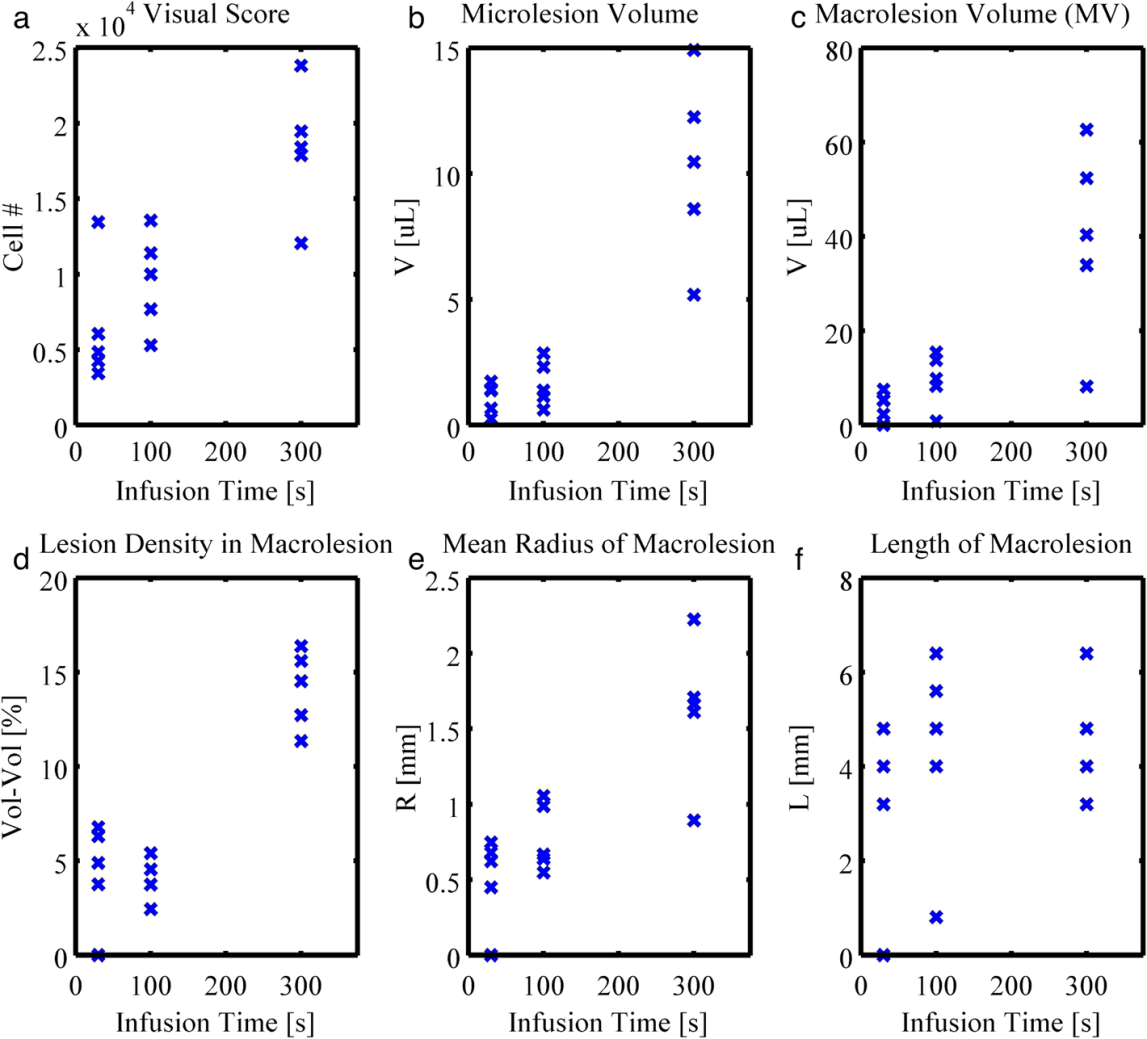
Scatter plot of characterized parameters versus infusion durations. Results for (**a**) visual score, (**b**) microlesion volume, (**c**) macrolesion volume, and (**d**) macrolesion mean radius show some linear dependence with respect to infusion duration, revealing the fact that the probability to create lesion is accumulative over time. Growth of the macrolesion radius (**e**) indicates that the macrolesion edges contribute to the lesion formation as well

The experiment of various infusion durations of contrast agents is potentially equivalent with longer duration exposure. As shown in Fig. 13, visual score, microlesion volume, macrolesion volume, and mean radius of macrolesion show positive correlation revealing the fact that the probability to create lesion is accumulated over time. Growth of the macrolesion radius indicates that the edge of the macrolesion is contributing to the lesion formation over time. Lesion density increases for longer infusion times. Short term and intermediate do not significantly differ. This could be due to the lack of statistical power or too short of a duration to allow for fully developed statistical analysis. The macrolesion lengths are not showing any strong correlation with the infusion interval, as they are likely limited by the distal ventricle and possibly due to tissue attenuation.

#### Microlesion density versus ultrasound amplitude

Least square fitting in Fig. 7a implies that an increasing acoustic pressure has a positive effect on microlesion formation up to 4 MPa, i.e., higher acoustic pressure or exposure possesses the higher potential to induce bioeffects. A similar positive correlation of acoustic amplitude on cavitation-induced bioeffects was also presented by Samuel et al. [18]. One possible reason is that larger pressure will result in a larger active microbubble population. Therefore, a larger density of microlesion will be induced by the then more frequent microbubble cavitation events. For a constant pressure amplitude, macrolesions also grow radially over time. This is because of the probabilistic accumulation of microlesions on the penumbra of the current macrolesion. There, the probability *χ* for microlesion generation is larger than 0 % and smaller than 100 %, i.e., the sound pressure amplitude is close to the threshold *p*_L_ discussed above. If *χ* is 20 %, then a five times longer exposure will statistically result in additional lesion formation.

#### Acoustic pressure threshold

The zero microlesion volume shown in the line fit in Fig. 7b might overestimate the acoustic pressure threshold for lesion induction. This is because microlesion volumes were characterized in a way that could bias towards beam regions with lower acoustic pressure and hence lower partial microlesion volume.

Additionally, the acoustic pressure threshold for microlesion induction by either lesion densities or microlesion volumes is a rough estimation and may be inaccurate due to biological variations, with *R*^2^ being 0.48 and 0.56.

### Application in human

Ideally, the wanted axial length of the transducer’s point spread function matches the myocardial thickness. Here in the presented small animal model, a shorter depth of field would have been desirable, though no side effects, except for some pulmonary hemorrhaging, presented in the study. The characterized macrolesions for all rats showed similar lengths, which in most cases here is due to the acoustic path restrained by the limited thickness of the left vertical wall. However, when the study moves to a larger animal model (such as swine) rather than the currently employed rodents (rats), the effect of therapeutic pressure on the macrolesion length will manifest itself and be vital for lesion formation and accretion.

The shown results serve as a preliminary test of MCET for application in humans. The main benefit from this therapy method is the minimal invasiveness and the hypothesis that cavitation-induced, sparsely distributed microlesions do not lead to major infarct-like scars, which can disrupt conduction pathways and lead to heart block, such as for alcohol ablation treatment [19]. The relationship between lesion characteristics from the small animal model, such as lesion dimension and density and in situ ultrasound field, is assumed to be analogous to that of a large animal model. Both models follow the same rationale of lesion formation. In the larger model, especially in humans, it is anticipated that we will have to create composite lesions, i.e., lesions created by electronically and/or mechanically sweeping the beam. For therapy of large volumes, the therapy beam will likely be scanned through the desired volume (simulated here by the Gaussian modulation) to accomplish the treatment in less time than is needed to treat point by point (as in HIFU). Suppose a human subject needs MCET treatment at a myocardial region of 4 cm diameter [20]. Assuming that the left ventricular wall of the hypertrophic heart has a 21-mm thickness [2], a macrolesion, approximated as a cylinder, with volume π × (4 cm/2)^2^ × 21 mm = 26.4 mL, would be needed. Assuming that the therapy employs the recommended dose of Definity® for diagnostic exams, i.e., 5 μL/kg/min, a 5-min treatment of a single focal spot will yield a 50-μL macrolesion with 20 % microlesion density. To create the aforementioned macrolesion, a total duration of approximately 2640 min will be needed to achieve the desired lesion volume. Recruitment of a swept beam to foster the lateral lesion formation at 56 fps, as discussed above, will reduce the total duration to 47 min. Stacking multiple axial focal zones will enlarge axial lesion size and further accelerate the therapy. The above calculations are under the assumption that the in vivo microbubble distribution in the myocardium is similar in human and the chosen rat model. A large animal model for human cardiophysiology, such as swine, will be needed for investigating the clinical transition for MCET.

## Conclusion

The quantitative scoring scheme overcomes the limitation of traditional visual scoring and works for histological cases with a large lesion count, i.e., has an appropriate dynamic range for evaluating therapeutic applications. The presented results have shown that MCET-induced macrolesions grow radially as the acoustic pressure amplitude increases. A swept beam as a new method to shorten treatment time seems promising but requires additional verification to ensure efficacy. These characterizations and validations may assist future MCET treatment planning.

## References

[1] Gersh BJ, Maron BJ, Bonow RO, Dearani JA, Fifer MA, Link MS (2011). 2011 ACCF/AHA guideline for the diagnosis and treatment of hypertrophic cardiomyopathy: executive summary: a report of the American college of cardiology foundation/American heart association task force on practice guidelines. J Am Coll Cardiol.

[2] Maron BJ, Maron MS (2013). Hypertrophic cardiomyopathy. Lancet.

[3] Marian AJ (2009). Contemporary treatment of hypertrophic cardiomyopathy. Tex Heart Inst J.

[4] Miller DL, Dou C, Owens GE, Kripfgans OD (2014). Optimization of ultrasound parameters of myocardial cavitation microlesions for therapeutic application. Ultrasound Med Biol.

[5] Miller DL, Dou CY, Lucchesi BR (2011). Are Ecg premature complexes induced by ultrasonic cavitation electrophysiological responses to irreversible cardiomyocyte injury?. Ultrasound Med Biol.

[6] Zhou C, Chan HP, Chughtai A, Patel S, Hadjiiski LM, Wei J (2012). Automated coronary artery tree extraction in coronary CT angiography using a multiscale enhancement and dynamic balloon tracking (MSCAR-DBT) method. Comput Med Imaging Graph.

[7] Bouraoui B, Ronse C, Baruthio J, Passat N, Germain P (2010). 3D segmentation of coronary arteries based on advanced mathematical morphology techniques. Comput Med Imaging Graph.

[8] Xu Y, Liang GY, Hu GS, Yang Y, Geng JZ, Saha PK (2012). Quantification of coronary arterial stenoses in CTA using fuzzy distance transform. Comput Med Imaging Graph.

[9] Zhou C, Hadjiiski LM, Sahiner B, Chan HP, Patel S, Cascade PN (2003). Computerized detection of pulmonary embolism in 3D computed tomographic (CT) images: vessel tracking and segmentation techniques. P Soc Photo Opt Ins.

[10] Bouma H, Sonnemans JJ, Vilanova A, Gerritsen FA (2009). Automatic detection of pulmonary embolism in CTA images. IEEE Trans Med Imaging.

[11] Moayyeri A, Adams JE, Adler RA, Krieg MA, Hans D, Compston J (2012). Quantitative ultrasound of the heel and fracture risk assessment: an updated meta-analysis. Osteoporosis Int.

[12] Pinter SZ, Rubin JM, Kripfgans OD, Treadwell MC, Romero VC, Richards MS (2012). Three-dimensional sonographic measurement of blood volume flow in the umbilical cord. J Ultrasound Med.

[13] Mamou J, Coron A, Hata M, Machi J, Yanagihara E, Laugier P (2010). Three-dimensional high-frequency characterization of cancerous lymph nodes. Ultrasound Med Biol.

[14] Zhu YI, Miller DL, Dou C, Kripfgans OD (2015). Characterization of macrolesions induced by myocardial cavitation-enabled therapy. IEEE Trans Biomed Eng.

[15] Miller DL, Dou Y, Lu F, Zhu YI, Fabiilli ML, Owens GE (2015). Use of theranostic strategies in myocardial cavitation-enabled therapy. Ultrasound Med Biol.

[16] Miller DL, Dou CY, Wiggins RC (2007). Simulation of diagnostic ultrasound image pulse sequences in cavitation bioeffects research. J Acoust Soc Am.

[17] Jensen JA, Svendsen NB (1992). Calculation of pressure fields from arbitrarily shaped, apodized, and excited ultrasound transducers. IEEE Trans Ultrason Ferroelectr Freq Control.

[18] Samuel S, Cooper MA, Bull JL, Fowlkes JB, Miller DL (2009). An ex vivo study of the correlation between acoustic emission and microvascular damage. Ultrasound Med Biol.

[19] ten Cate FJ, Soliman OI, Michels M, Theuns DA, de Jong PL, Geleijnse ML (2010). Long-term outcome of alcohol septal ablation in patients with obstructive hypertrophic cardiomyopathy: a word of caution. Circ Heart Fail.

[20] Maron BJ (2005). Surgery for hypertrophic obstructive cardiomyopathy: alive and quite well. Circulation.

